# Unified Embedding and Composable Fusion for Arbitrary Multimodal Person Re-Identification

**DOI:** 10.3390/s26206395

**Published:** 2026-10-10

**Authors:** Wenchao Gao, Sen Song, Yifan Chen, Yue Xin, Xiaodan Li

**Affiliations:** School of Artificial Intelligence, China University of Mining and Technology-Beijing, Beijing 100083, China; 18831035112@163.com (S.S.); cyf08132024@163.com (Y.C.); x165614@163.com (Y.X.); 13772535312@163.com (X.L.)

**Keywords:** multimodal person re-identification, feature enhancement, feature fusion, unified embedding representation

## Abstract

Driven by the advancement of intelligent security, person re-identification has evolved from single-modality imagery to complex multimodal tasks involving infrared, text, sketches, and paintings. However, most existing methods are restricted to fixed modality pairs and struggle to accommodate the flexible fusion requirements of variable inputs, making the integration of arbitrary modality combinations a critical challenge. To address this, we propose the Unified Embedding Representation Fusion Network (UERF-Net), a framework based on unified embedding representation fusion. This method first establishes a unified embedding space to map heterogeneous modalities into a shared representation, effectively narrowing the modality gap through an explicit pre-alignment strategy. It then introduces a composable fusion mechanism utilizing a joint self-attention module to facilitate deep token-level interactions across diverse modality combinations. Furthermore, a distribution alignment strategy is employed to optimize similarity distributions via Supervised Distribution Alignment (SDA), significantly enhancing feature stability and identity discriminative power. Experimental results on the five-modality ORBench benchmark demonstrate that UERF-Net achieves state-of-the-art performance in Rank-1 accuracy (R1) and mean average precision (mAP), confirming the robustness and superior adaptability of our unified embedding-based modeling in complex, multi-source retrieval scenarios.

## 1. Introduction

From a sensor-level perspective, earlier studies demonstrated the value of far-infrared cameras and image fusion for robust person detection under challenging imaging conditions [1]. Such work provides practical evidence for exploiting complementary information from heterogeneous sensing sources in pedestrian analysis.

Person Re-identification (ReID) [2] is a quintessential task in computer vision aimed at searching for the same pedestrian identity across large-scale surveillance or multi-camera networks [3], which requires accurate differentiation between individuals among pedestrians with minimal inter-class variations and similar appearances. Due to its high application value in scenarios such as urban security and smart transportation, it has received extensive attention from researchers [4]. Early ReID methods [5] primarily relied on handcrafted features and metric learning models, such as color-texture descriptors [6] and Large Margin Nearest Neighbor (LMNN) [7]; however, these methods exhibited limited robustness under complex conditions like illumination changes, occlusions, and pose variations. With the advancement of deep learning, ReID has gradually transitioned toward end-to-end representation learning, significantly enhancing feature discrimination through objective functions such as classification loss [8], triplet loss [9], and contrastive loss [10]. Simultaneously, representative methods based on human part-based modeling [11] further strengthened the representation of local details. Although traditional ReID primarily utilizes visible images as queries, in real-world surveillance and security applications—such as nighttime monitoring, occlusion, long-distance capture, or missing image records—the query information acquired at the front end may only consist of infrared images, or even natural language descriptions, manual sketches, or paintings, while the gallery usually remains dominated by visible imagery. Among these, visible-infrared person re-identification (VI-ReID) [12] is the most typical category, which utilizes infrared queries to retrieve visible images [13]. With the development of natural language processing [14], the text modality has enabled the abstract description of pedestrian clothing, attributes, and behavioral characteristics from a semantic level [15], thereby compensating for the deficiencies of visual modalities under low-illumination and occluded conditions [16]. Textual information demonstrates significant value, particularly when pedestrian images are incomplete or when retrieval must rely solely on witness descriptions [17].

However, with the continuous evolution of sensing devices and multi-source information acquisition methods, query information in real-world security scenarios has exhibited increased diversity. Query conditions provided by users may consist of various combinations of modalities such as infrared, sketch, painting, and text. In this context, how to map heterogeneous query information from different sources and expressions into a unified feature space and perform effective joint modeling has become a core problem in multimodal person re-identification research. Strong complementary relationships exist among different modalities. Relying solely on a single modality or limited modality combinations often leads to insufficient identity representation due to imaging discrepancies, occlusions, and information loss. Consequently, an increasing number of multimodal datasets aligned with real-world application scenarios have been proposed [18], shifting the research focus from simple dual-modality mapping toward unified representation learning of multi-source heterogeneous features. Conducting research on multimodal person re-identification possesses clear practical significance, the core of which lies in exploring how to coordinate shared and modality-specific information within a unified embedding space to learn more discriminative identity representations under variable modality combinations.

To address the limitations of existing ReID methods, which are often restricted to fixed modality pairs and struggle to adapt to the flexible fusion requirements of varied query combinations, we aim to construct a unified framework capable of integrating arbitrary modality inputs. The primary challenge of this task lies in bridging the representation gap among diverse data sources—including visible light, infrared, text, sketch, and colored painting—and extracting stable identity features under highly uncertain query conditions. Consequently, as a solution for complex retrieval requirements, we have explored a feature learning strategy based on unified embedding and dynamic fusion.

**Figure 1:**
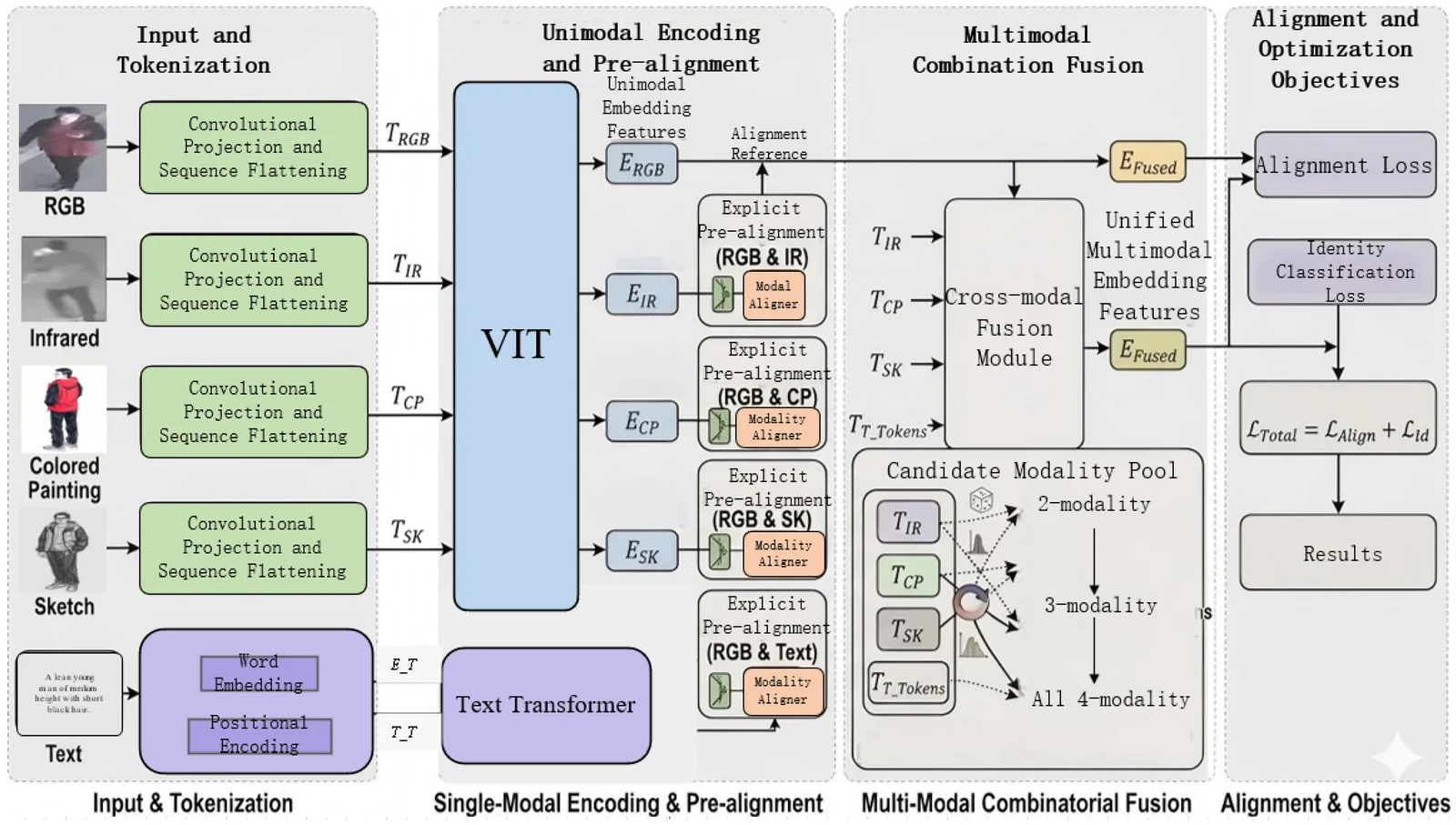
The UERF-Net structure. Solid arrows indicate the main feature-processing flow, dashed arrows indicate modality selection and combination, and the star denotes the final fused representation used for optimization.

To this end, we propose UERF-Net, a unified embedding representation fusion framework. This method maps heterogeneous modalities into a shared representation and effectively narrows the modality gap through an explicit pre-alignment strategy. We designed a systematic modeling pipeline consisting of tokenization, pre-alignment, and a core combinational fusion stage, which leverages a joint self-attention mechanism to facilitate deep token-level interactions. Under this architecture, the model adaptively handles arbitrary combination inputs ranging from unimodal to quadmodal. Rather than treating Contrastive Language–Image Pre-training (CLIP) or self-attention as independently novel components, UERF-Net contributes a coordinated framework that integrates unified representation, explicit pre-alignment, composable fusion, and supervised distribution alignment for variable multimodal queries. As shown in Figure 1, which is introduced in detail in Section 3.1, UERF-Net maintains robust identity discriminative power under variable combinations of available query information. Our main contributions are summarized as follows:

- We present a unified embedding and alignment framework for person re-identification, i.e., UERF-Net, which leverages the cross-modal prior of CLIP [19] to bridge the representation gap among heterogeneous data. The framework maps five heterogeneous modalities into a unified space through an explicit pre-alignment strategy and unimodal identity constraints, establishing a structural foundation for deep multi-source synergy.
- We devise a composable fusion module for arbitrary queries based on Joint Self-attention mechanism. Addressing the challenge of uncertain modality combinations in practical applications, this mechanism enables deep token-level interaction, allowing the model to adaptively generate highly discriminative composite features for any given modality combination.
- We introduce an SDA optimization strategy to enhance the robustness of fused features in complex scenarios. By enforcing consistency between the joint feature distribution and the reference modality distribution, this strategy ensures optimization stability while significantly strengthening the discriminative power of multi-modal representations at the identity level.
- Extensive experiments on the ORBench dataset [20] have demonstrated that UERF-Net achieves leading performance in handling multi-source heterogeneous tasks. Notably, our method reaches state-of-the-art (SOTA) levels in R1 and mAP, exhibiting superior adaptability and robustness in multi-modal combinatorial retrieval tasks.

## 2. Related Work

In recent years, the research scope of ReID has gradually expanded from traditional single-modality visible light to multimodal tasks. To address the challenges posed by multi-source heterogeneous information, existing research has primarily focused on the following two connected directions:

### 2.1. Current Status of Cross-Modal Person Re-Identification

The fundamental challenge of VI-ReID lies in the significant modality gap and distribution shift caused by cross-spectral imaging [21]. Existing VI-ReID methods can be broadly categorized into image-level and feature-level approaches. Image-level methods often employ generative adversarial networks (GANs) for visible-to-infrared translation or style transfer [22], aiming to align appearance styles while preserving identity information as much as possible. Among mainstream feature-level methods, the cross-modality shared-specific feature transfer algorithm (cm-SSFT) [23], proposed by Lu et al., explicitly models shared and specific features and explores their complementary relationship through cross-modal feature transfer. Hierarchical Cross-Modality Disentanglement (Hi-CMD) [24], introduced by Choi et al., adopts a hierarchical decoupling strategy to separate identity-related factors from modality-related factors. Building on these, the Modality Confusion Learning Network (MCLNet) [25] learns modality-inseparable identity representations via modality mixing and center aggregation. Additionally, the works of Feng et al. [26] and Wei et al. [27] have respectively leveraged orthogonal decomposition and discriminant enhancement to further tap into the complementarity between different feature components, thereby reducing the interference of modality-related factors on identity discrimination.

Meanwhile, the advancement of text-image person re-identification [28] has demonstrated that natural language descriptions can serve as effective semantic supplements for pedestrian retrieval. The core idea of text-aided cross-modal re-identification [29] is to introduce text as a supplementary cue within the infrared-visible matching framework to mitigate the scarcity of visual information. A representative work is YYDS [30], proposed by Du et al., which utilizes coarse-grained linguistic descriptions alongside infrared images as queries to assist in visible-light target retrieval. Subsequently, focusing on the exploitation of generative semantics, TVI-LFM [31] by Hu et al. leverages text descriptions generated by Vision-Language Models (VLM) and enhanced by Large Language Models (LLM) to enrich the representation capability of the infrared modality. This shift signifies that the text modality has evolved from a purely external auxiliary source into an essential component capable of deep involvement in feature learning and representation completion, offering a new evolutionary path for cross-modal person re-identification.

### 2.2. Current Status of Multimodal Unified Retrieval and Dynamic Fusion

In the field of multimodal research, unified representation learning and joint retrieval modeling of multi-source diverse information have become the research focus. TriReID [32] is one of the early representative works explicitly oriented toward multimodal ReID, proposing to jointly utilize cues such as images, sketches, and text for identity retrieval via a description fusion model. Subsequently, Chen et al. [33] pointed out that users may not always provide fixed types of descriptive queries, necessitating research into a unified retrieval framework for different description modalities. Building on this, the All-in-One (AIO) framework [34] simultaneously processes multiple cues—such as visible, infrared, sketches, and text—to meet multimodal retrieval needs in wild environments. Currently, multimodal person re-identification is moving toward more modalities and more flexible query combinations. ORBench, a five-modality benchmark for omni multimodal person ReID, and ReID5o, its associated unified retrieval model [20], were proposed under such research requirements. ORBench explicitly divides testing into four search patterns—single, dual, triple, and quadruple-modality queries—prompting current research to emphasize shared representation learning within a unified embedding space and dynamic fusion mechanisms for diversified query combinations.

Although these methods provide important foundations, they emphasize different aspects of multimodal retrieval. UNIReID [33] mainly unifies different descriptive-query tasks through task-specific modality learning and task-aware dynamic training, whereas AIO [34] employs modality tokenization, a shared pretrained encoder, and cross-modality heads to accommodate multiple retrieval tasks. ReID5o [20] further extends unified retrieval to five modalities and their different combinations. In comparison, UERF-Net emphasizes the coordinated relationship among three stages: heterogeneous inputs are represented in a shared CLIP space and explicitly pre-aligned toward an RGB-centered identity space; the available modality tokens are then compositionally fused to support arbitrary unimodal-to-quadmodal queries; and the fused representations are jointly constrained by identity supervision and SDA. Therefore, the primary contribution of UERF-Net lies in integrating explicit pre-alignment, flexible token-level interaction, and distribution-level supervision into a continuous pipeline for variable multimodal queries.

## 3. Multimodal Person Re-Identification Method Based on Unified Embedding Representation Fusion

The proposed method aims at achieving multimodal person re-identification under uncertain and complex query conditions. To bridge the modality gap, we construct a unified embedding space based on CLIP cross-modal priors and an explicit pre-alignment strategy. As for information integration, we propose a composable fusion module utilizing joint self-attention to facilitate deep interactions across arbitrary modality combinations. Through the combination of SDA and identity supervision, UERF-Net can effectively exploit multi-source complementarity and ensure identity discriminative power. The architecture of the proposed method is shown in Figure 1.

### 3.1. Model Architecture

To address the challenges of highly uncertain modality combinations and difficult multi-source information fusion in real-world security scenarios, this chapter proposes UERF-Net, a multimodal person re-identification method based on unified embedding representation fusion. CLIP is a vision–language pre-training model with separate visual and text encoders. Through contrastive learning on large-scale paired image–text data, it maps matched images and texts into a shared semantic embedding space [19]. This cross-modal prior provides semantically compatible initial representations, but it does not by itself eliminate the distribution differences among RGB, infrared, colored-painting, sketch, and text modalities in person re-identification.

Accordingly, UERF-Net initializes the four visual branches from the CLIP Vision Transformer (ViT) encoder, specifically the ViT-B/16 variant, and employs the CLIP text encoder for the text modality. Modality-specific Low-Rank Adaptation (LoRA) is then used to retain the pretrained knowledge while accommodating modality-dependent visual characteristics, and explicit pre-alignment further reduces the residual representation shift before multimodal fusion. The model follows a systematic pipeline of “tokenization, pre-alignment, dynamic fusion, and joint optimization”. First, image modalities are mapped to unified-dimension visual tokens, while text features are extracted to eliminate initial structural discrepancies. Subsequently, a shared-weight ViT backbone [35] extracts shared identity semantics and performs explicit pre-alignment centered on the RGB modality to bridge the representation gap. On this basis, the multimodal combination fusion module utilizes a joint self-attention mechanism derived from the standard Transformer attention formulation [36] to facilitate deep token-level interactions for arbitrary modality combinations, ensuring robustness against missing information. Finally, by combining SDA and identity classification losses, the model achieves cross-modal consistency and identity discriminability through end-to-end training. This structural design enables stable and accurate identity matching under complex and variable retrieval conditions.

### 3.2. Multimodal Input and Tokenization

In the model input phase, this paper receives five types of data: red–green–blue (RGB) visible images, infrared (IR) images, colored painting (CP) images, sketch (SK) images, and text descriptions. Considering the significant differences in imaging mechanisms between visual and language modalities, the model constructs separate visual and text input branches to preserve the original characteristics of each modality while establishing a foundation for subsequent unified representation learning.

**Figure 2:**
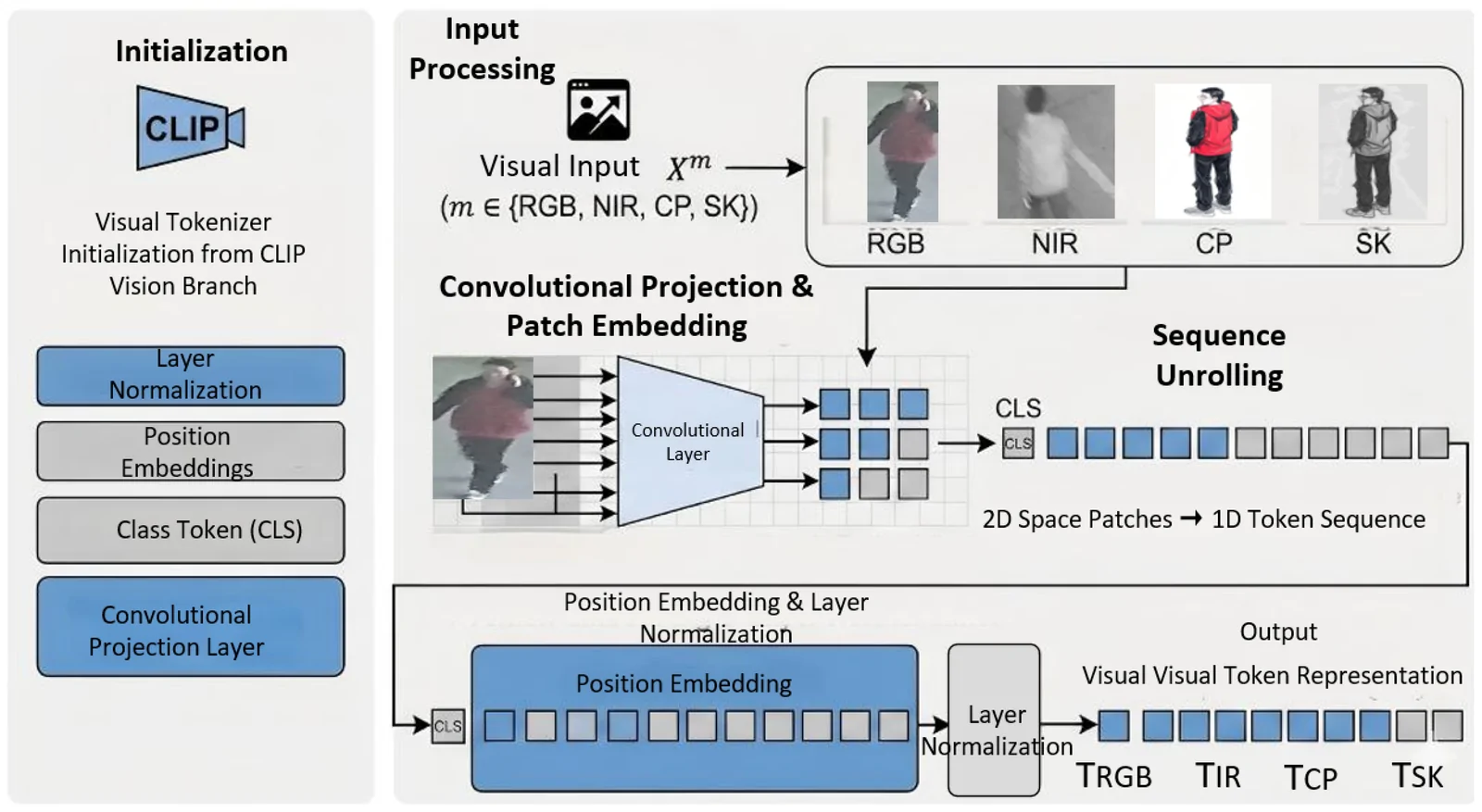
Visual Modality Input and Tokenization. The blue and grey blocks are used only to distinguish token positions visually and do not represent different processing branches.

For visual modalities, independent visual tokenizers initialized with the CLIP visual branch are constructed for RGB, IR, CP, and SK. For any visual input $X^{i}$, the model first partitions the image into local patches via a convolutional projection layer and maps them into embedding representations. Subsequently, the features are flattened into a 1D token sequence, where a global class (CLS) token is concatenated to the prefix and position encodings are added. After Layer Normalization, the serialized input representations are obtained. The tokenized outputs for the four visual modalities are denoted as $T_{RGB},T_{IR},T_{CP}$ and $T_{SK}$, respectively, as illustrated in Figure 2.

Through this process, all image modalities are converted into token sequences, enabling natural integration with the Transformer encoding framework. Meanwhile, the original text is tokenized and mapped into continuous word vector sequences containing positional information via an embedding layer. Consequently, the text modality is also represented as a sequence input $T_{T}$ for Transformer modeling. Ultimately, the visual and text modalities achieve a unified input format, providing a reliable basis for subsequent multi-modal joint modeling based on token-level concatenation.

### 3.3. Single-Modality Encoding and Alignment

The tokenized visual modalities are fed into a unified ViT encoder [35], which learns $E_{RGB}$, $E_{IR}$, $E_{CP}$ and $E_{SK}$ within a unified embedding space through multi-layer self-attention mechanisms. This approach aims to extract shared identity semantics, such as body contours and clothing structures, via a unified backbone. Compared to ResNet50, ViT more effectively captures cross-regional structural relationships due to its global modeling capability. Furthermore, the encoder incorporates the LoRA mechanism introduced by Hu et al. [37], utilizing lightweight low-rank branches to enhance modality-specific adaptation while preserving pre-trained knowledge, thus balancing shared semantic learning with the requirements of differentiated modeling. Specifically, LoRA branches are inserted into the query–key–value projection, attention output projection, and two feed-forward linear layers of all 12 visual Transformer blocks. Four modality-specific LoRA parameter sets are used, with rank r=4, scaling coefficient α/r=1/4, and dropout set to 0.

**Figure 3:**
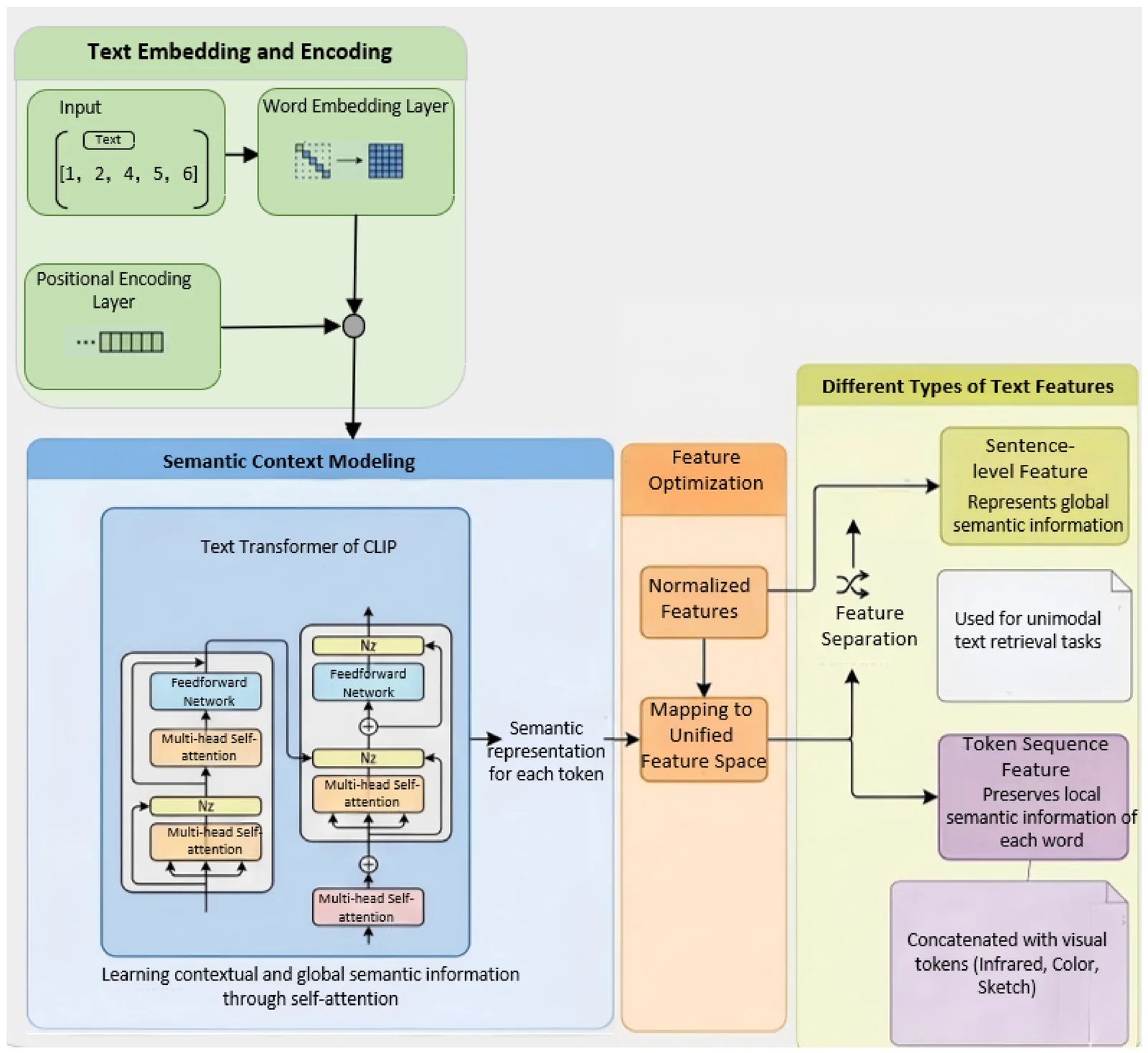
Text Modality Encoding Architecture.

The text modality employs an independent CLIP text encoder to model contextual dependencies and projects the features into the unified space. The encoder outputs two levels of features: the sentence-level global feature $E_{T}$ for macro-semantic representation, and the token sequence feature $T_{T}$ for fine-grained local information. This dual-level feature design provides a stable global vector while establishing a foundation for the deep synergy of multi-source heterogeneous information. The corresponding text encoding architecture is illustrated in Figure 3.

In summary, the global embeddings $E_{RGB}$, $E_{IR}$, $E_{CP}$, $E_{SK}$, and $E_{T}$ obtained during the unimodal encoding phase form the basis for subsequent modeling. For a batch containing *B* samples, $E_{m}={\left\{e_{m}^{i}\right\}}_{i=1}^{B}\in R^{B\times d}$ denotes the set of *d*-dimensional global embeddings for modality m∈{RGB,IR,CP,SK,T}. For the visual modalities, $e_{m}^{i}$ is the projected CLS representation produced by the ViT encoder; for text, $e_{T}^{i}$ is the sentence-level global representation produced by the CLIP text encoder. To mitigate representation shift and enhance cross-modal consistency, a pre-alignment strategy is introduced using $E_{RGB}$ as the reference. Let $l_{\mathrm{align}}\left(E_{a},E_{b}\right)$ denote a binary alignment-loss function that measures the discrepancy between the two sets of modality embeddings $E_{a}$ and $E_{b}$. In the final UERF-Net configuration, $l_{\mathrm{align}}$ is instantiated by the SDA loss defined in Section 3.5. The unimodal pre-alignment objective is therefore defined as

(1) $$L_{\mathrm{pre}}=l_{\mathrm{align}}\left(E_{RGB},E_{IR}\right)+l_{\mathrm{align}}\left(E_{RGB},E_{CP}\right)+l_{\mathrm{align}}\left(E_{RGB},E_{SK}\right)+l_{\mathrm{align}}\left(E_{RGB},E_{T}\right).$$

### 3.4. Multimodal Fusion Module

**Figure 4:**
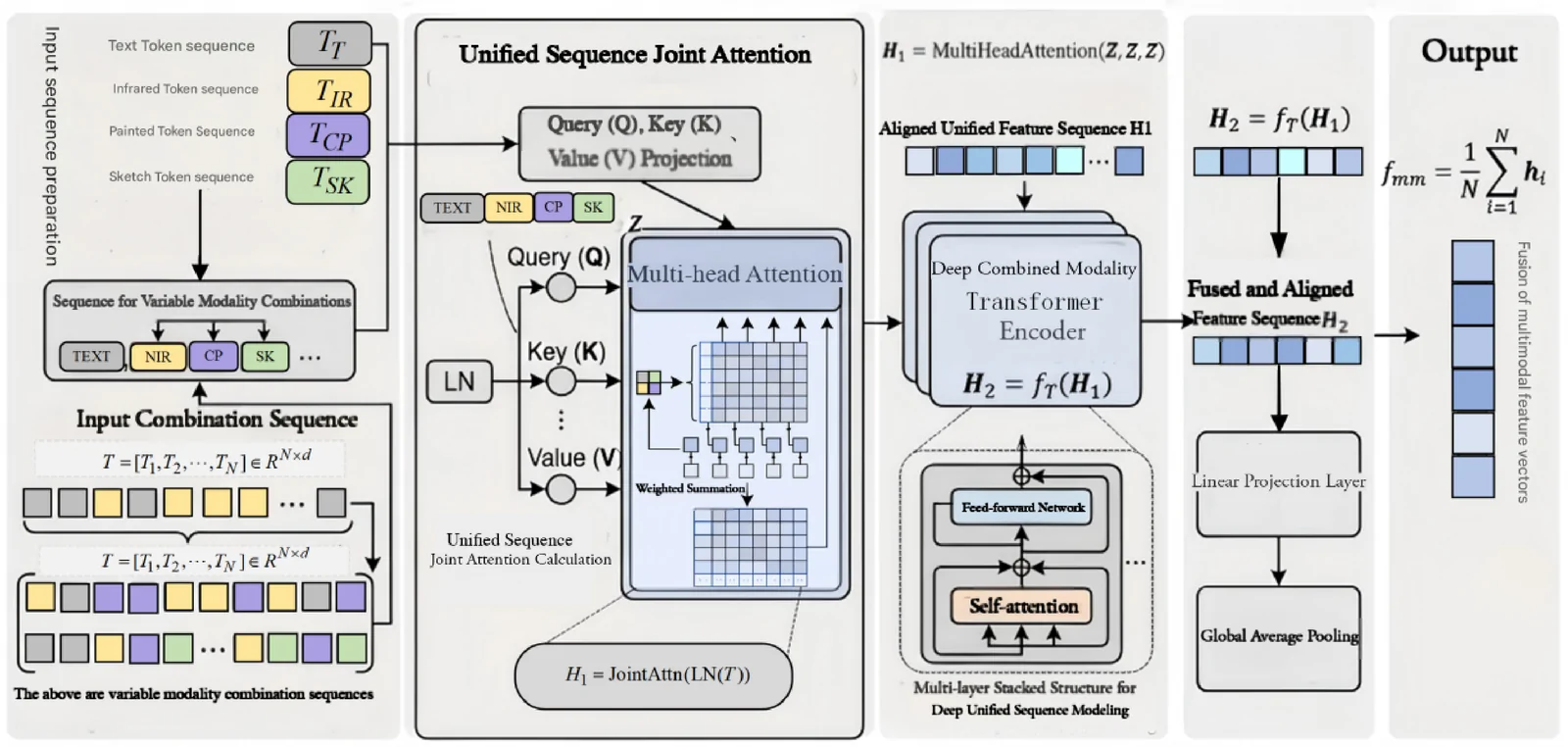
Multimodal Fusion Module Architecture.

Once multimodal combinations are constructed, heterogeneous modality token sequences are concatenated to form the input sequence. Since this concatenation merely organizes information at the structural level without achieving deep semantic fusion, the model introduces a cross-modal fusion module based on joint self-attention to perform semantic correlation modeling within a unified feature space. The detailed process is illustrated in Figure 4.

First, in the input preparation stage, the model selects token sequences from the candidate pool consisting of infrared, colored painting, sketch, and text, and concatenates them along the token dimension to construct a unified multimodal joint input sequence representation:

(2) $$T=\left[T_{1},T_{2},\ldots ,T_{N}\right]\in R^{N\times d}$$

Here, *N* and *d* denote the total number of tokens and the embedding dimension, respectively. As a joint representation containing multi-source heterogeneous semantics, *T* can uniformly process arbitrary combinations of inputs ranging from single modality to quadruple modalities, providing a standard interface for subsequent modeling. On this basis, the model performs joint self-attention computation on the sequence *T*: first, Layer Normalization (LN) [38] is applied to mitigate the numerical distribution differences across modalities; then, following the standard self-attention formulation [36], the sequence is projected into the Query (*Q*), Key (*K*), and Value (*V*) matrices, and attention weights are computed across the entire sequence to establish inter-modal dependencies. The symbols *Q*, *K*, and *V* are used exclusively for these attention matrices; query samples and ground-truth distributions in the loss functions are denoted using different symbols. This process can be expressed as

(3) $$H_{1}=\mathrm{JointAttn}\left(LN\left(T\right)\right)$$

where $H_{1}$ represents the output feature sequence after the initial interaction. At this point, each token in $H_{1}$ is capable of perceiving and absorbing relevant information from other modalities within the unified attention space.

To further explore high-order dependencies, the interaction feature $H_{1}$ is fed into a deep composite modality Transformer encoder $f_{T}\left(\cdot \right)$ [36] for further modeling:

(4) $$H_{2}=f_{T}\left(H_{1}\right)$$

where $H_{2}$ represents the fused feature sequence after deep modeling. In the implementation, all modality tokens are projected to d=512. The initial joint-attention module is standard eight-head self-attention, with a head dimension of 64; its query, key, and value are generated from the same layer-normalized concatenated sequence. No modality-specific attention mask is applied, so every token can attend to all tokens from the available modalities. The attention output is subsequently processed by one Transformer residual block (mmt_depth=1), which contains pre-layer normalization, eight-head self-attention, and a two-layer feed-forward network with dimensions 512→2048→512, QuickGELU activation, and residual connections. The attention and feed-forward dropout rates are both set to 0. This stage enhances the complementarity of information across different modalities, allowing the tokens of each modality to form a more discriminative joint representation within the unified semantic space. Finally, a projection head consisting of a 512→512 linear layer, QuickGELU, layer normalization, and a second 512→512 linear layer adjusts the output space. Global average pooling [39] is then performed on the $H_{2}$ sequence to obtain a multimodal fusion feature vector of unified dimension:

(5) $$f_{mm}=\frac{1}{N}\sum_{i=1}^{N}h_{i}$$

where $h_{i}$ is the feature of the *i*-th token, and $f_{mm}$ integrates information from local structure, global semantics, and cross-modal relationships. As the core representation for subsequent alignment with visible light features, $f_{mm}$ significantly improves cross-modal consistency within the unified embedding space.

### 3.5. Loss Function Design

Following the completion of cross-modal fusion, an optimization objective consisting of cross-modal alignment loss and identity classification loss is designed to establish consistent semantic correspondences between query features and visible light targets in the unified embedding space. The model supports five types of loss functions: SDA, Picture–Text Contrastive (PT), Supervised Picture–Text Contrastive (SPT), Cross-Modal Projection Matching (CMPM), and Identity Classification (ID) Loss, with SDA and ID Loss serving as the core objectives.

(1) SDA Loss

SDA operates at the feature alignment stage, aiming to establish consistent matching relationships between query features and visible light target features in the shared space under the supervision of identity labels. Its distribution-matching term follows the Similarity Distribution Matching (SDM) formulation introduced by Jiang and Ye [40], which is extended here from a fixed image–text pair to arbitrary multimodal query combinations with cardinality-balanced supervision. Let M={IR,CP,SK,T} denote the set of query modalities and let $C_{k}=\left\{S\subseteq M:|S|=k\right\}$ denote the collection of modality subsets containing *k* modalities. For a batch of *B* samples and a query subset $S\in C_{k}$, the predicted query-to-reference similarity distribution is defined as

(6) $$p_{ij}^{S\to R}=\frac{\exp\;\left(\mathrm{sim}(z_{S}^{i},z_{R}^{j})/\tau \right)}{\sum_{n=1}^{B}\exp\;\left(\mathrm{sim}(z_{S}^{i},z_{R}^{n})/\tau \right)},$$

where $z_{S}^{i}$ and $z_{R}^{j}$ are the fused query feature and RGB reference feature, respectively, and τ is the temperature parameter. The reverse distribution $p_{ij}^{R\to S}$ is obtained by exchanging the query and reference roles. The identity-supervised target distribution is

(7) $$g_{ij}=\frac{1(y_{i}=y_{j})}{\sum_{n=1}^{B}1\left(y_{i}=y_{n}\right)},$$

which assigns probability mass to all samples sharing the same identity rather than to only one paired sample. For each modality subset *S*, the bidirectional distribution-alignment loss is

(8) $$L_{S}^{\mathrm{SDA}}=\frac{1}{B}\sum_{i=1}^{B}\sum_{j=1}^{B}\left[p_{ij}^{S\to R}\log\frac{p_{ij}^{S\to R}}{g_{ij}+\epsilon }+p_{ij}^{R\to S}\log\frac{p_{ij}^{R\to S}}{g_{ij}+\epsilon }\right],$$

where ϵ is a small constant for numerical stability. Finally, SDA balances the supervision of different query cardinalities by averaging the losses within each subset size before summing the four cardinality levels:

(9) $$L_{\mathrm{SDA}}=\sum_{k=1}^{4}\frac{1}{|C_{k}|}\sum_{S\in C_{k}}L_{S}^{\mathrm{SDA}}.$$

This formulation preserves the identity-supervised similarity-distribution matching principle of SDM while extending it to variable multimodal query subsets. It also prevents a cardinality level with more possible modality combinations from dominating the optimization, enabling the model to jointly accommodate unimodal and complex multimodal retrieval scenarios.

(2) Identity Classification Loss

To enhance the discriminative capability and class separability at the identity level, explicit identity supervision is applied to unimodal and fused global features $f\in R^{d}$, where *d* denotes the feature dimension. An identity prediction score vector $\hat{y}$ is obtained through linear classification mapping:

(10) $$\hat{y}=Wf+b$$

where *W* and *b* represent the weights and bias of the classification layer, respectively. Subsequently, the standard softmax cross-entropy objective [41] is calculated using the ground-truth identity class $c^{★}$:

(11) $$L_{id}=-\sum_{i=1}^{C}1\left(i=c^{★}\right)\log\frac{\exp({\hat{y}}_{i})}{\sum_{j=1}^{C}\exp\left({\hat{y}}_{j}\right)}$$

where *C* represents the total number of identity classes in the training set and $1(i=c^{★})$ is the indicator function. Specifically, it takes the value of 1 when the *i*-th class is the ground-truth identity category and 0 otherwise. The objective of this loss is to increase the prediction probability of the correct identity class and suppress the responses of other categories, thereby enhancing the discriminability of features at the class level.

(3) Auxiliary Contrastive and Projection Losses

This section comprises the PT loss, the SPT loss, and the CMPM loss, aiming to further reinforce the consistency of the unified embedding space through multi-dimensional constraints.

PT adopts the temperature-scaled contrastive formulation established by InfoNCE [42] and subsequently used in CLIP [19] to pull positive sample pairs closer and push negative sample pairs further apart. Let $f_{\mathrm{ref}}$ denote the visible light reference features and $f_{\mathrm{qry}}$ denote the query features. The similarity is calculated using the temperature parameter τ:

(12) $$s_{ij}=\frac{f_{\mathrm{ref},i}^{T}f_{\mathrm{qry},j}}{∥f_{\mathrm{ref},i}{∥}_{2}{∥f_{\mathrm{qry},j}∥}_{2}\tau }$$

The corresponding image-to-query contrastive loss $L_{PT}$ is expressed as

(13) $$L_{PT}^{\mathrm{ref}\to \mathrm{qry}}=-\frac{1}{B}\sum_{i=1}^{B}\log\frac{\exp(s_{ii})}{\sum_{j=1}^{B}\exp\left(s_{ij}\right)}$$

Following supervised contrastive learning [43], SPT further incorporates identity labels to construct the same-identity positive index set S(i), encouraging features of the same identity under different combinations to aggregate into adjacent regions:

(14) $$L_{SPT}=-\sum_{i}\frac{1}{\left|S(i)\right|}\sum_{p\in S(i)}\log\frac{\exp(\mathrm{sim}\left(f_{i},f_{p}\right)/\tau )}{\sum_{a\neq i}\exp\left(\mathrm{sim}\left(f_{i},f_{a}\right)/\tau \right)}$$

CMPM follows the Cross-Modal Projection Matching formulation introduced by Zhang and Lu [44] and achieves projection alignment through distribution-level constraints. By calculating the projection similarity $P_{ij}$ between the query feature $f_{\mathrm{qry},i}$ and the visible light reference feature ${\hat{f}}_{\mathrm{ref},j}$:

(15) $$P_{ij}=f_{\mathrm{qry},i}^{T}{\hat{f}}_{\mathrm{ref},j}$$

Ultimately, by constraining the predicted distribution to remain consistent with the ground-truth distribution $G_{ij}$, the loss $L_{CMPM}$ is represented as:

(16) $$L_{CMPM}=\sum_{i}\sum_{j}{\hat{P}}_{ij}\left(\log{\hat{P}}_{ij}-\log G_{ij}\right)$$

(4) Global Optimization Objective

The optimization objective of this paper is to simultaneously achieve cross-modal feature alignment and identity discriminative enhancement within a unified embedding space. The cross-modal alignment loss constrains the semantic consistency between query features of different modalities (and their combinations) and the visible light target features, bringing representations of the same identity closer; meanwhile, the identity classification loss enhances the class separability of features, ensuring that different identities maintain clear boundaries. The total optimization objective can be formulated as

(17) $$L=L_{SDA}+\lambda L_{id}$$

where λ is a balance coefficient used to adjust the weight of identity classification supervision in the overall optimization. In all experiments, λ is set to 1.0.

Furthermore, let the SDA losses between unimodal, bimodal, trimodal, and quadruple-modal queries and the visible light target features be $L_{SDA}^{\left(1\right)}$, $L_{SDA}^{\left(2\right)}$, $L_{SDA}^{\left(3\right)}$ and $L_{SDA}^{\left(4\right)}$, respectively; the overall objective can be further expressed as

(18) $$L=L_{SDA}^{\left(1\right)}+L_{SDA}^{\left(2\right)}+L_{SDA}^{\left(3\right)}+L_{SDA}^{\left(4\right)}+\lambda L_{id}$$

This form indicates that the model integrates query representations of different scales (from unimodal to quadruple-modal) into a single optimization framework. This ensures that each query mode can be mapped to a consistent visible light target space while maintaining stable identity discriminability.

## 4. Experiments

### 4.1. Re-Ranking Strategy

Considering that the chunk-based re-ranking strategy can more effectively distinguish the structural relationships among intra-query domain, intra-gallery domain, and cross-domain matching in complex cross-modal retrieval scenario—thereby reducing interference caused by the mixed propagation of different types of neighborhood information—this study adopts this method for experimental evaluation. Specifically, to address the more complex feature distribution relationships between multimodal joint queries and the visible-light gallery, this paper follows the approach of modeling different relationship sub-blocks separately followed by comprehensive optimization. This strategy post-processes the initial distance matrix to further enhance the accuracy and stability of the final retrieval results.

### 4.2. Experimental Datasets and Parameter Settings

This paper employs the representative dataset ORBench. This dataset encompasses five modalities: visible light, infrared, colored painting, sketch, and text. We selected ORBench [20] because its identity-aligned five-modality composition directly matches the RGB-gallery retrieval setting considered in this study. It permits systematic evaluation of all $2^{4}-1=15$ non-empty query subsets formed by infrared, colored painting, sketch, and text, from unimodal to quadmodal retrieval. In contrast, many widely used cross-modal ReID datasets are designed for fixed modality-pair protocols and thus cannot evaluate a single framework under arbitrary modality availability; ORBench’s manually annotated colored paintings and semantically rich text also provide challenging heterogeneous cues. Therefore, ORBench provides an appropriate unified protocol for a fair comparison with existing omni-modal methods, while the limitation of single-benchmark validation and the need for future cross-dataset evaluation are explicitly discussed in the Discussion section.

The experiments utilize a pre-trained CLIP ViT-B/16 as the unified backbone. The maximum length of the text branch is set to 77, and the vocabulary size is 49,408. All input images are resized to 384×128. During training, random horizontal flipping with a probability of 0.5, 10-pixel padding followed by random cropping, and random erasing with a probability of 0.5 and an area ratio of 0.02–0.4 are applied. For the text input, 15% of valid tokens are selected for augmentation; among them, 80% are replaced by the mask token, 10% by a random token, and 10% remain unchanged. The training batch size is 16, and the model is trained for 60 epochs using mixed-precision training. Adam is adopted with $\beta_{1}=0.9$, $\beta_{2}=0.999$, $\epsilon =10^{-8}$, and a weight decay of $4\times 10^{-5}$. The base learning rate is $1\times 10^{-5}$, whereas the newly initialized fusion layers, classifier, and LoRA parameters use a five-times learning rate of $5\times 10^{-5}$. A five-epoch linear warm-up is used, starting from 0.1 times the corresponding learning rate, after which the learning rate is reduced to 0 by cosine decay over the remaining epochs. Finally, R1 and mAP, standard metrics in the field of person re-identification, are employed to measure retrieval accuracy and ranking quality.

### 4.3. Experimental Results and Analysis

#### 4.3.1. Result Analysis

In terms of baseline selection, the proposed method is compared with various representative cross-modal person re-identification methods and general multimodal frameworks. To ensure the stability and fairness of the comparison, the experimental results for each modality in the table are not derived from a single individual test. Instead, they represent the average performance statistics across different query conditions and combination scenarios under the corresponding modality setting. The specific results are presented in Table 1:

#### 4.3.2. Computational Cost and Inference Efficiency

To evaluate the computational cost of UERF-Net under different query configurations, we report the total parameter count, average floating-point operations (FLOPs), inference latency, and throughput in Table 2. The FLOPs and inference results for each configuration are averaged over all modality combinations containing the corresponding number of modalities.

As the number of query modalities increases, the computational cost grows because additional modality encoders are activated and longer joint token sequences are processed by the fusion module. Nevertheless, even in the most computationally demanding quadmodal setting, UERF-Net processes a query in 38.83 ms and maintains a throughput of 25.75 samples/s. These results demonstrate that the composable design provides flexible multimodal fusion with a predictable increase in computational cost as more information sources become available.

#### 4.3.3. Ablation Study

This section analyzes the contribution and dominance of various modalities by examining the experimental results across different combinations. First, comparative experiments were conducted on the four single modalities in the query set, with specific results presented in Table 3.

Under unimodal retrieval conditions, the CP modality achieved the highest R1 and mAP metrics, indicating its superior discriminative power and dominant role within the current multimodal retrieval framework.

To explore the impact of different modal combinations as query inputs, multiple sets of comparative experiments were designed to calculate the average performance of each category across Rank-1 and mAP indicators.

As observed in Table 4 and Table 5, the Rank-1 and mAP metrics generally follow an upward trend as the number of retrieval modalities increases. Trimodal combinations typically outperform bimodal ones, verifying that multimodal joint modeling effectively integrates complementary information from diverse sources. This synergy compensates for the limitations of individual modalities, thereby enhancing the completeness of feature representation and the stability of retrieval ranking. These results also prove that the proposed fusion framework possesses strong adaptability and scalability under varying modal counts.

From the perspective of specific combinations, the contribution of the CP modality is the most significant. Experiments demonstrate that combinations including CP consistently outperform those without it; for example, NIR + CP significantly surpasses NIR + SK and NIR + TEXT.

Furthermore, the experiments reveal how complementarity affects performance: if information redundancy between modalities is high, the resulting bottleneck persists even in more complex retrieval tasks. Specifically, CP + SK emerged as the worst-performing bimodal combination because CP and SK are highly similar in human contours and structural information while lacking fine-grained appearance details. This information overlap leads to the trimodal performance of CP + SK + TEXT remaining lower than that of CP + NIR + TEXT, which benefits from higher complementarity.

Finally, the bimodal visualization results further validate these conclusions: combinations containing CP show excellent performance, whereas combinations involving the TEXT modality generally yield lower results, reflecting the inherent differences in identity discriminative capability among the different modalities.

#### 4.3.4. Visualization Analysis

(1) Convergence Analysis

**Figure 5:**
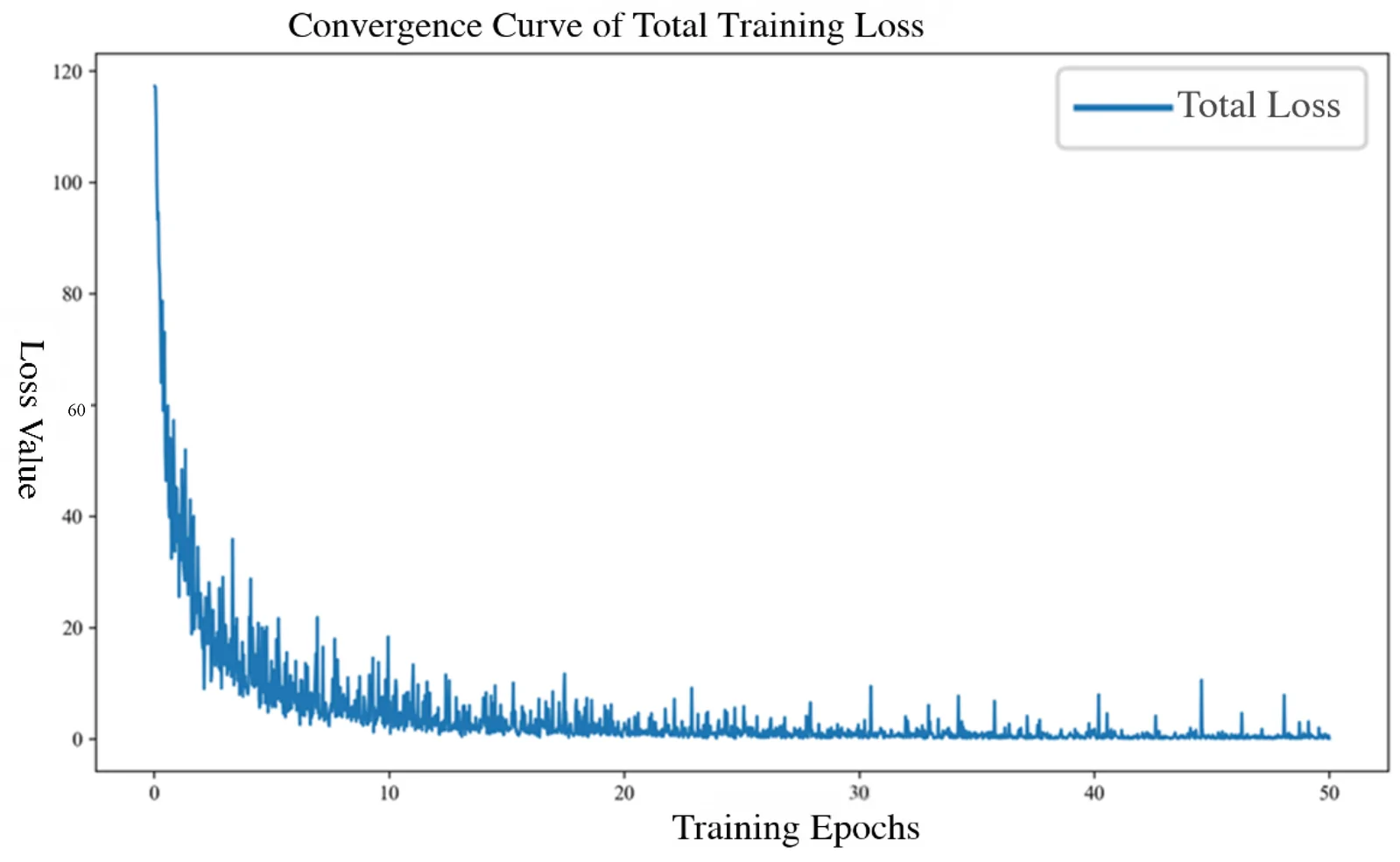
Total Loss Convergence Curve.

**Figure 6:**
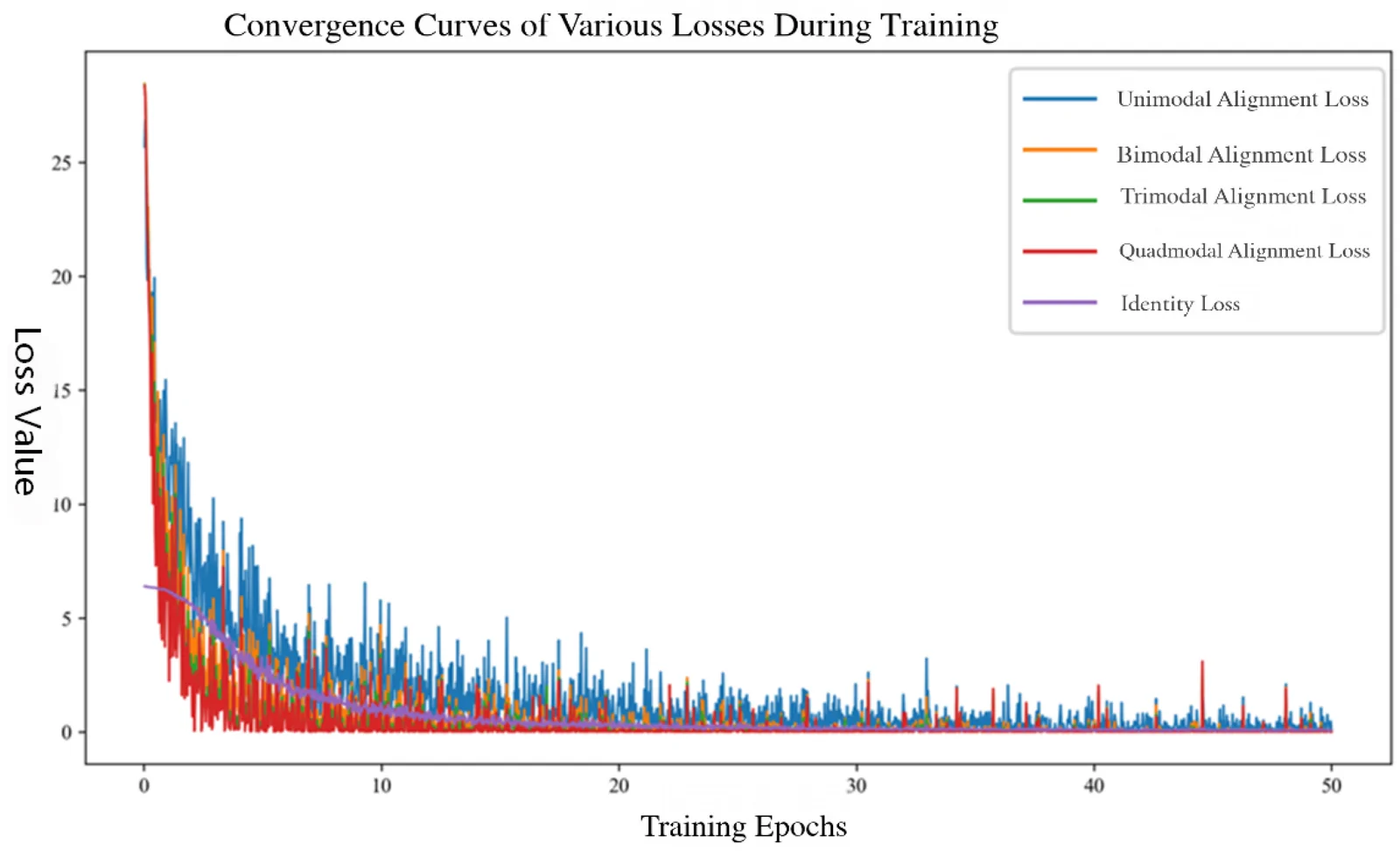
Loss Convergence Curves.

To further verify the optimization stability and convergence characteristics of the proposed method during the training process, this paper performs a visualization analysis of the total loss and its individual components during training. Figure 5 and Figure 6 present the corresponding convergence curves, respectively.

As shown in Figure 5 and Figure 6, each loss component decreases rapidly in the early stages of training and eventually stabilizes at a low level, indicating that the network can quickly learn discriminative information and possesses good convergence. The synchronous stabilization of the alignment loss and identity loss powerfully demonstrates the robustness of the proposed method in balancing cross-modal consistency and identity discriminative learning.

(2) Qualitative Retrieval Results

**Figure 7:**
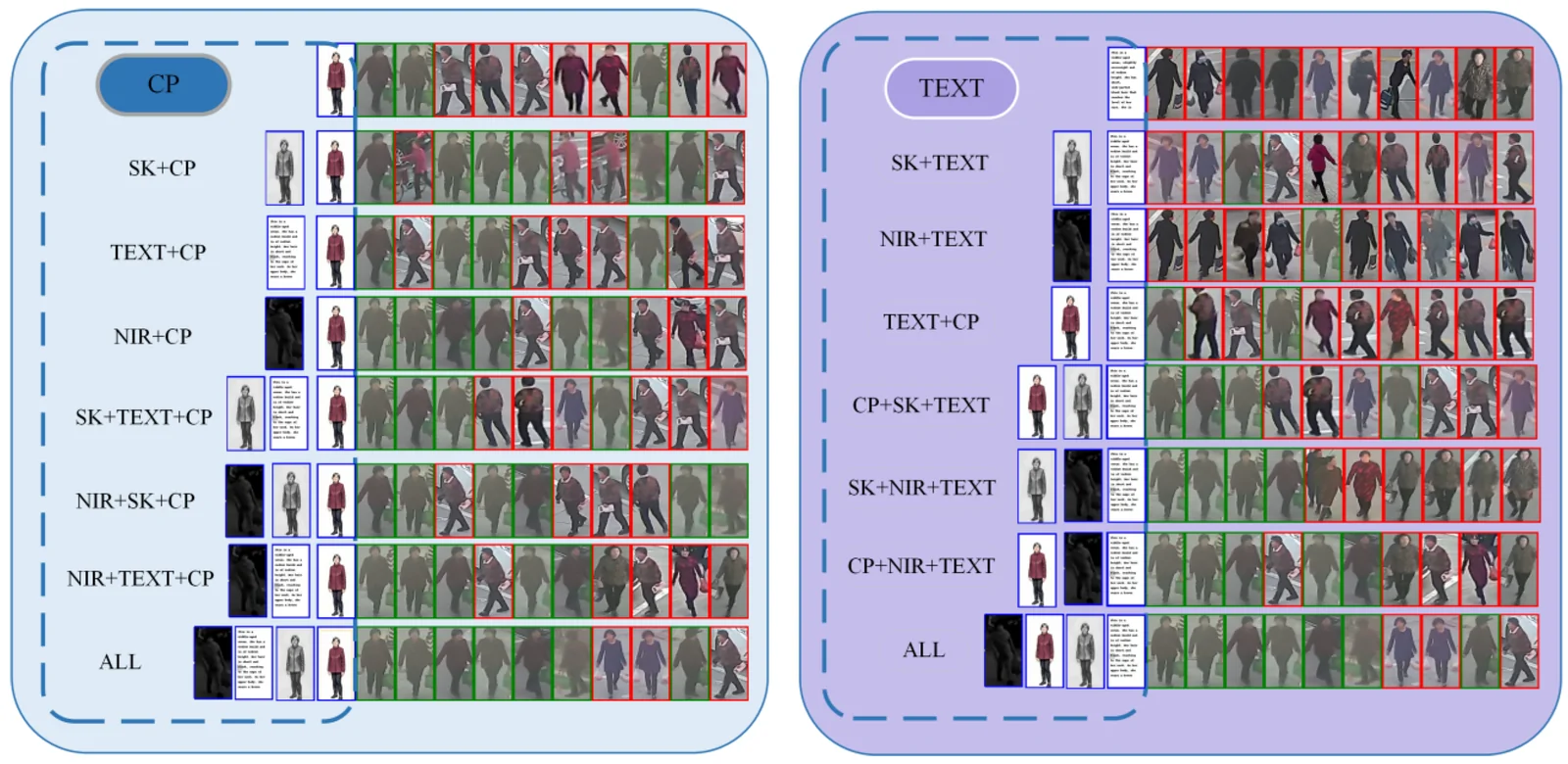
Visualization of Retrieval Results for Text and Painted Modalities. Blue boxes indicate query inputs, green boxes indicate correct retrievals, and red boxes indicate incorrect retrievals.

As shown in Figure 7, the visualization analysis of retrieval results reveals that, as the number of query modalities increases, the number of correctly matched samples significantly grows and their distribution becomes more stable, verifying that multimodal fusion effectively integrates complementary information and enhances retrieval robustness. Furthermore, unimodal comparisons show that the colored painting modality outperforms text in discriminative ability due to intuitive visual cues, confirming its dominant role in identity representation and the auxiliary value of text as a semantic supplement.

#### 4.3.5. Comparative Experiments on Loss Functions

In this experiment, comparative analyses were conducted for four alignment losses: SDA, PT, SPT, and CMPM. By replacing the loss function within the same multimodal framework, this study aims to evaluate the impact of each loss on model performance. The experimental configurations and results are summarized in Table 6.

The final results demonstrate that the SDA loss achieves the best performance across all experimental settings, significantly enhancing the model’s cross-modal alignment performance and identity classification accuracy. The primary reason is that SDA effectively captures the similarity distribution between different modalities. By maximizing inter-modal similarity for the same identity and minimizing the distance between different identities, SDA achieves more precise cross-modal alignment. Furthermore, the ID loss further strengthens the model’s discriminative power, ensuring that the model not only establishes robust alignment across modalities but also maintains strong separability for identity determination.

In contrast, the PT loss essentially employs contrastive learning based on one-to-one sample correspondence within a batch. It assumes that each sample forms a unique positive pair only with the feature of another modality at the same index, treating all other samples as negatives. While this mechanism can pull paired samples closer, its constraint granularity is biased toward single-point matching. When facing complex shared identity relationships, it tends to erroneously treat same-identity samples—which should be proximal—as negatives. Although SPT advances beyond PT by alleviating the over-constrained nature of single-positive samples, its fundamental objective remains the enhancement of discriminability (pulling positives and pushing negatives) rather than directly fitting the cross-modal similarity distribution structure. Finally, the CMPM projection constraint is highly sensitive to the shape of the feature space distribution. After merging multimodal combination features, the statistical discrepancies between different combinations become more complex. Consequently, CMPM is more susceptible to fluctuations in projection scales and local similarities, leading to an optimization objective that is less stable than SDA. Therefore, the combination of SDA and ID loss has been proven to be the most effective optimization strategy.

To verify the effectiveness of the proposed fusion strategy and further analyze the impact of different feature fusion methods on experimental performance, comparative experiments were designed for various fusion strategies. While keeping the remaining network architecture, training settings, and loss functions consistent, we compared feature mean fusion, feature weighted fusion, max pooling fusion, and the proposed fusion strategy. The comparative results are summarized in Table 7.

Specifically, feature mean fusion performs element-wise averaging of the CLS tokens from each modality. Although structurally simple, this approach tends to weaken the complementary information between modalities. Weighted fusion assigns learnable weights to different modalities, enabling the model to adaptively adjust their contributions. However, the fusion remains predominantly at the global vector level. By prematurely compressing the representations into a single global vector, substantial local details are lost, and the lack of explicit fine-grained interaction between modalities makes it difficult to fully exploit their complementarity. Max pooling fusion selects the strongest response in each dimension across the fused sequence features to highlight local salient information; nevertheless, it overemphasizes extreme values while neglecting the overall complementary relationships among modalities.

In contrast, the proposed method preserves the token-level sequence representations of each modality. It achieves preliminary information interaction via a joint attention mechanism, followed by deep Transformer layers to iteratively model cross-modal relationships. Finally, a sequence aggregation method is employed to obtain more comprehensive unified fused features. Therefore, this set of ablation experiments intuitively demonstrates the discrepancies in information utilization capabilities among different fusion methods in multimodal scenarios, validating the advantages of the proposed strategy in fine-grained interaction modeling and overall retrieval performance enhancement.

## 5. Discussion

The experimental results indicate that UERF-Net can effectively handle variable multimodal query configurations on ORBench. The modality analysis suggests that the colored-painting modality provides strong standalone identity cues, whereas text has lower standalone discriminability but supplies useful semantic information when combined with visual inputs. The relatively weaker CP + SK results also show that adding modalities does not necessarily improve retrieval when their information is highly redundant. This observation supports the use of token-level joint attention and deeper Transformer interaction, which allow the model to exploit complementary information before global aggregation. In addition, the loss comparison indicates that SDA improves retrieval by supervising similarity distributions rather than relying only on sample-level contrastive relationships.

More specifically, text-only queries may confuse visually similar pedestrians when coarse semantic descriptions lack fine-grained identity attributes, while the relatively weaker CP + SK performance indicates that combining two visually abstract modalities with redundant cues does not necessarily improve retrieval. Even with multiple modalities, identity ambiguity can remain when the available cues are insufficiently complementary; therefore, the proposed fusion improves robustness but cannot eliminate all difficult matches.

These results also reveal practical limitations and challenging cases. The current evaluation is based on ORBench only, and the model has not been systematically tested with severely noisy inputs, extreme modality missingness, or deployment on edge and resource-constrained sensing platforms. Future work will therefore evaluate cross-dataset generalization, robustness to corrupted or incomplete modalities, and more efficient fusion mechanisms for practical multimodal sensing and retrieval systems.

## 6. Conclusions

This study addressed person re-identification under arbitrary combinations of heterogeneous query modalities. UERF-Net integrates unified embedding learning, RGB-centered pre-alignment, composable token-level fusion, and supervised distribution alignment into a single end-to-end framework. The experiments on ORBench achieved average R1/mAP values of 69.55%/60.29%, 85.61%/75.76%, 93.05%/83.58%, and 95.82%/87.06% for unimodal, bimodal, trimodal, and quadmodal queries, respectively, demonstrating the effectiveness of the proposed framework for flexible multimodal retrieval.

The present study is limited to ORBench and does not yet systematically evaluate severe modality corruption, extreme missing information, cross-dataset generalization, or deployment on resource-constrained sensing platforms. These aspects will be investigated in future work to further improve the robustness and practical efficiency of multimodal person re-identification systems.

## Figures and Tables

**Table 1:** Experimental Comparison Results of Different Methods.

Method	Unimodal Average		Bimodal Average		Trimodal Average		Quadmodal Average	
	R1 (%)	mAP (%)	R1 (%)	mAP (%)	R1 (%)	mAP (%)	R1 (%)	mAP (%)
Meta-TF [45]	6.25	4.24	10.81	6.68	14.82	8.65	18.32	10.45
AIO [34]	50.44	40.14	58.82	49.34	69.72	58.58	75.33	63.48
UNIReID [33]	59.49	47.98	80.91	66.83	88.71	74.86	91.34	78.12
CLIP [19]	59.84	48.73	80.31	65.79	87.67	73.17	90.71	76.69
ImageBind [46]	60.18	48.61	81.78	67.02	88.73	74.20	91.05	77.36
PLIP [47]	60.57	48.79	80.78	65.63	88.14	74.60	90.95	77.87
RDE [48]	60.91	48.82	82.36	67.52	89.94	75.27	92.75	78.64
IRRA [40]	61.17	51.42	82.50	68.83	89.70	75.86	92.65	79.15
**UERF-Net**	**69.55**	**60.29**	**85.61**	**75.76**	**93.05**	**83.58**	**95.82**	**87.06**

Note: Bold values indicate the best result in each column. The results in the table represent the average values of various modality combinations and their corresponding experimental results in different orderings, intended to comprehensively reflect the overall performance of each method across various combination scenarios.

**Table 2:** Computational Cost and Inference Efficiency of UERF-Net under Different Query Configurations.

Query Configuration	No. of Combinations	Params (M)	GFLOPs	Latency (ms)	Throughput (Samples/s)
Unimodal	4	159.24	27.75	9.28	107.72
Bimodal	6	159.24	59.06	20.91	47.82
Trimodal	4	159.24	88.90	29.93	33.41
Quadmodal	1	159.24	118.97	38.83	25.75

**Table 3:** Retrieval Results for Single-Modal Query Tasks.

Unimodal	R1 (%)	mAP (%)
Near-infrared (NIR)	57.56	48.79
Text	63.56	55.22
SK	71.31	61.15
CP	85.76	75.99

**Table 4:** Retrieval Results for the Dual-Modal Combined Query Task.

Bimodal	R1 (%)	mAP (%)
NIR + TEXT	81.23	71.06
SK + TEXT	80.88	71.38
NIR + SK	85.15	74.80
CP + SK	85.25	74.50
CP + TEXT	87.67	78.16
NIR + CP	93.46	84.67

**Table 5:** Retrieval Results for Query Tasks under Trimodal Combination.

Trimodal	R1 (%)	mAP (%)
CP + SK + TEXT	89.81	79.62
NIR + SK + TEXT	92.63	82.80
NIR + CP + SK	94.61	85.26
NIR + CP + TEXT	95.13	86.63

**Table 6:** Experimental Results of Different Alignment Loss Functions.

Loss Function	R1 (%)	mAP (%)
PT	90.47	82.64
SPT	93.56	86.52
CMPM	95.17	86.47
**SDA**	**95.82**	**87.06**

Note: Bold values indicate the best result in each column.

**Table 7:** Comparison of Different Rearrangement Strategies.

Method	Unimodal		Bimodal		Trimodal		All-Modal	
	R1 (%)	mAP (%)	R1 (%)	mAP (%)	R1 (%)	mAP (%)	R1 (%)	mAP (%)
Feature Average	35.51	28.69	42.27	42.19	52.72	45.55	55.34	46.22
Feature Weighted	38.12	30.09	46.15	45.26	53.71	48.12	56.52	48.42
Max Pooling	64.61	55.29	81.72	70.45	85.45	79.15	88.23	82.21
**Proposed Method**	**69.55**	**60.29**	**85.61**	**75.76**	**93.05**	**83.58**	**95.82**	**87.06**

Note: Bold values indicate the best result in each column.

## Data Availability

The ORBench dataset supporting the findings of this study is openly available in accordance with standard academic benchmarks.

## Abbreviations

The following abbreviations are used in this manuscript:

UERF-Net	Unified Embedding Representation Fusion Network
CLIP	Contrastive Language–Image Pre-training
cm-SSFT	Cross-Modality Shared-Specific Feature Transfer
Hi-CMD	Hierarchical Cross-Modality Disentanglement
MCLNet	Modality Confusion Learning Network
AIO	All-in-One
ORBench	Five-Modality Omni Multimodal Person Re-Identification Benchmark
ReID5o	Omni Multi-Modal Person Re-Identification in a Single Model
LoRA	Low-Rank Adaptation
NIR	Near-Infrared
SK	Sketch
CP	Colored Painting
SDA	Supervised Distribution Alignment
SDM	Similarity Distribution Matching
PT	Picture-Text
SPT	Supervised Picture-Text
CMPM	Cross-Modal Projection Matching
CLS	Class Token
R1	Rank-1
mAP	Mean Average Precision
